# The Effect of a Virtual Reality Delivered Physical Activity Intervention on the Physical Function of Older Adults

**DOI:** 10.1093/geroni/igab046.3638

**Published:** 2021-12-17

**Authors:** Kyle Kershner, Joy Furlipa, Peter Brubaker, Amber Brooks, Lindsey Page, Diane Ehlers, Jason Fanning

**Affiliations:** 1 University of Nebraska Medical Center, Omaha, Nebraska, United States; 2 Wake Forest University, Winston-Salem, North Carolina, United States; 3 Department of Anesthesiology, Wake Forest School of Medicine, North Carolina, United States

## Abstract

COVID-19 public health recommendations have prohibited many older adults from attending in-person physical activity (PA) programs that improve physical function and promote functional independence. Most PA programs have shifted towards a video conference (VC) format, but this modality has been noted to “flatten” the social experience which is fundamental for lasting behavior change. Virtual reality (VR) is now designed for immersion and place-presence and may be better suited for instilling a feeling of social connection, which will likely improve physical function. The purpose of this study was to evaluate differences in physical function after a 4-week in-home VR or VC based PA intervention. Low-active adults (66.8±4.8 years) were randomized to VR (n=5) or VC (n=4) based PA counselling and instructed to find activities that were intrinsically motivating. VR participants were asked to select pre-approved available active games in addition to enjoyable real-world activities. ANCOVA models were used to explore group differences in six-minute walk distances across time. Results are reported using η^2 effect sizes based on the small sample size. After controlling for baseline values, the ANCOVA models revealed a moderate-to-large magnitude effect for distance traveled during the six-minute walk test (η^2=.10). Additionally, the VR group participants walked 42.63 meters further, which approaches a clinically meaningful difference. These promising early findings suggest there is value to exploring the impact of VR-delivered, group-mediated activity promotion on physical functioning in older adults. Future research should investigate aspects of VR that promote increased social connection and physical function in the older adult population.

